# Prevalence of attention deficit/hyperactivity disorder among children and adolescents in China: a systematic review and meta-analysis

**DOI:** 10.1186/s12888-016-1187-9

**Published:** 2017-01-19

**Authors:** Tingting Wang, Kaihua Liu, Zhanzhan Li, Yang Xu, Yuan Liu, Wenpei Shi, Lizhang Chen

**Affiliations:** 1 Department of Epidemiology and Health Statistics, Xiangya School of Public Health, Central South University, NO. 238 Shang Ma Yuan Ling Xiang Xiangya Road, Kaifu District, Changsha, Hunan Province China; 2 Department of Toxicology, Xiangya School of Public Health, Central South University, Changsha, Hunan Province China; 3 Department of Oncology, Xiangya Hospital, Central South University, Changsha, Hunan Province China; 4 Deyang Center for Disease Control and Prevention, Sichuan Province, China

**Keywords:** Attention deficit/hyperactivity disorder, Prevalence, China, Meta-analysis

## Abstract

**Background:**

Attention deficit/hyperactivity disorder (ADHD), the most common childhood neurobehavioural disorder, can produce a series of negative effects on children, adolescents, and even adults as well as place a serious economic burden on families and society. However, the prevalence of ADHD is not well understood in China. The goal of this study was to estimate the pooled prevalence of ADHD among children and adolescents in China using a systematic review and meta-analysis.

**Methods:**

A systematic literature search was conducted in PubMed, Web of Science, MEDLINE, CNKI, Wanfang, Weipu and CBM databases, and relevant articles published from inception to March 1, 2016, that provided the prevalence of ADHD among children and adolescents in China were reviewed. The risk of bias in individual studies was assessed using the Risk of Bias Tool for prevalence studies. Pooled-prevalence estimates were calculated with a random-effects model. Sources of heterogeneity were explored using subgroup analyses.

**Results:**

Sixty-seven studies with a total of 275,502 individuals were included in this study. The overall pooled-prevalence of ADHD among children and adolescents in China was 6.26% (95% CI: 5.36–7.22%) with significant heterogeneity (I^2^ = 99.0%, *P* < 0.001). The subgroup analyses showed that, the variables “geographic location” and “source of information” partially explained of the heterogeneity in this study (*P* < 0.05). The prevalence of ADHD-I was the highest of the subtypes, followed by ADHD-HI and ADHD-C.

**Conclusions:**

The prevalence of ADHD among children and adolescents in China is generally consistent with the worldwide prevalence and shows that ADHD affects quite a large number of people under 18 years old. However, a nationwide study is needed to provide more accurate estimations.

**Electronic supplementary material:**

The online version of this article (doi:10.1186/s12888-016-1187-9) contains supplementary material, which is available to authorized users.

## Background

Attention deficit/hyperactivity disorder (ADHD), the most common neurobehavioural disorder in childhood, is characterized by inattention, hyperactivity, impulsivity, low frustration tolerance, and a lack of organizational behaviour disproportionate to age [1]. The DSM-IV [2] (Diagnostic and Statistical Manual of Mental Disorders, 4th edition) divided ADHD into three subtypes: attention deficit (ADHD-I), impulsivity (ADHD-H) and mixed type (ADHD-C). Compared with typically developing children, children with ADHD have poorer interpersonal, parent-child and sibling relationships and lower academic achievement, resulting in a lack of self-esteem, low self-evaluation, negative emotions and other negative effects [3, 4]. ADHD was once considered a disease that was limited to childhood [5], but in past decades, this perspective has been gradually changed by evidence from a number of cross-sectional studies and retrospective studies, especially multi-centre follow-up studies. Researchers began to realize that in addition to children, ADHD also affects teenagers and even adults [6–9]. Symptoms of ADHD in children lasted into adolescence. From childhood to adolescence, hyperactivity symptoms were reduced, but attention deficits and impulsive symptoms remained, complicating interpersonal relationships and adversely affecting family relationships [10]. ADHD primarily affects learning ability [11, 12], antisocial behaviour [13], the incidence of traffic accidents [14] and the incidence of sex problems [15, 16] (e.g., early pregnancy, early sexual behaviour, sexual crime) in adolescents. During adulthood, ADHD patients face many problems in educational status, occupational function, family and interpersonal relationships [6, 8, 9], and even antisocial personality disorders and substance abuse [6–9, 17]. As a result of the diverse negative effects on patients and the serious economic burden on the families and society [18], ADHD has become a major public health concern [19].

In the past decade, several systematic reviews have been conducted to calculate the prevalence estimates of ADHD. According to a recent meta-analysis conducted with 175 eligible studies across the world, the prevalence of ADHD among children and adolescents is 7.2% (95%CI: 6.7–7.8%) [20], suggesting that a vast number of children and adolescents worldwide suffer from ADHD and that this widespread prevalence has led to a substantial burden on society.

In the past 30 years, an increasing number of scholars have been committed to the epidemiological study of ADHD in China. However, the prevalence rates reported in existing studies were limited to certain areas and showed a large variation. For example, the prevalence in individuals aged 7–16 years in Guiyang City was 0.73% [21], whereas in individuals aged 3–6 years in Nanjing City, it was 2.50% [22], in individuals aged 3–6 years in Guangzhou City, 4.83% [23], in individuals aged 5–13 years in Lanzhou City, 9.09% [24], in individuals aged 7–16 years in Liaoyang City, 11.50% [25], and in individuals aged 5–6 years in Shenzhen City, 14.40% [26]. Therefore, it is important to analyse the data provided in previous epidemiological studies using integrated methods, as this could provide a better understanding of the epidemic status and characteristics of ADHD among all children and adolescents.

Accordingly, the main purpose of the present systematic review and meta-analysis is to estimate the prevalence rates of ADHD among children and adolescents in China and to explore the possible causes of the inconsistencies in the reported rates of the included studies.

## Methods

This systematic review and meta-analysis adheres to PRISMA guidelines.

### Search strategy

We searched the PubMed, Web of Science, MEDLINE, China National Knowledge Infrastructure (CNKI), Wanfang, Weipu and China Biology Medicine disc (CBM disc) databases from inception to March 1, 2016, for articles in English and Chinese. The following search terms were used: minimal brain dysfunction, attention deficit disorder with hyperactivity, attention deficit, hyperactivity, hyperkinesis, MBD, ADHD, epidemiology, prevalence, rate, children, and adolescent. In addition, a manual search was performed of the reference lists of all articles selected in the first step. The entire process was independently completed by two researchers.

### Inclusion criteria and exclusion criteria

The inclusion criteria were as follows: 1) original investigations reporting data on ADHD among children (under 12 years old) and adolescents (12–18 years old) in China; 2) diagnostic criteria including the CCMD (Chinese Classification of Mental Disorders) (2, 2-R, 3), ICD (International Classification of Diseases) (9 or 10) or DSM (III, III-R, IV, 5); 3) samples obtained from the general population or schools by a probability sampling method; 4) information about prevalence estimates; and 5) cross-sectional studies or the first evaluation of longitudinal studies. The exclusion criteria were as follows: 1) studies that did not reporting the prevalence of ADHD or information adequate to evaluate the prevalence; 2) studies using clinical settings as the sample source; 3) studies on adults or special populations (e.g., juvenile offenders, students with internet addiction disorder or immigrant Chinese-American children); 4) quantitative studies, case-control studies, editorials, case reports and reviews; 5) studies with incomplete or unclear data or logical errors; and 6) duplicate publications and studies using the same data sources. In addition, if the same data were published in both English and Chinese, the paper published in Chinese was excluded.

### Data extraction

Using a study-designed protocol, two researchers extracted and evaluated the information from all included studies. They extracted the data from full-text articles separately, and a third researcher reviewed the data. Disagreements were resolved via discussion and expert consultation. The following information was extracted from each article: first author, year of publication, geographic location, study design, origin of sample (school or general population), subjects (children and adolescents), age range or mean age, source of information (e.g., subjects, clinicians, parents or teachers), assessment tools, clinical interview (yes or no), diagnostic criteria, sample size, and total prevalence estimate of ADHD.

Our primary purpose was to analyse the trends in ADHD prevalence rates with the time of assessment. However, 32.84% (22/67) of the studies did not report the year of assessment, and assessment year was thus replaced by the year of publication.

To calculate the pooled prevalence, we extracted only one ADHD prevalence estimate from each study. With reference to the practice of Thomas [20], the most conservative diagnosis was used in studies reporting more than one estimate. Previous studies reported a lower prevalence of ADHD among children and adolescents when children/adolescents were the informants compared with when their parents were informants [27, 28], and when parents compared with teachers were the informants [27, 29], and clinicians were demonstrated to report the lowest prevalence of all informants [27, 30]. Hence, if a study reported more than one estimate from different informants, we first considered the clinicians’ estimate, then the children’s, parents’, and finally the teachers’. If a study reported multiple estimates over time in the same sample, we chose the first one.

Some studies used more than one informant to identify those under 18 who were having ADHD symptoms. To clarify the source of information, we used the following categories: “and rule” (positive if endorsed by two or more informants), “or rule” (positive if endorsed by either teachers or parents), and “clinicians” (positive if endorsed by clinicians in a clinical interview).

### Assessment of risk of bias

The risk of bias in individual studies was assessed by using the Risk of Bias Tool for prevalence studies which was developed by Hoy et al. [31]. The tool was composed of ten items assessing the risk of bias in the following domains: selection bias (items 1–3), non-response bias (item 4), measurement bias (items 5–9) and bias related to the meta-analysis (item 10). One of the ten items (item 6) required studies to use an acceptable case definition. As we only included studies using the DSM, ICD or CCMD as the diagnostic criteria, this item was considered to be irrelevant to our study and was thus ruled out. For each criterion, the risk of bias was assessed as “low risk” or “high risk”. If the text was unclear, a “high risk” was recorded. The more criteria were met in the included studies, the lower the risk of bias. A study was rated as having a low risk of bias if seven or more items were met, a moderate risk of bias if 5 to 6 items were met, and a high risk of bias if four or fewer items were met [32].

### Statistical analysis

Before calculating the pooled prevalence, we performed a normality test for the original study rates and for the transformed rates, which were transformed using Log, Logit, arcsine and Freeman-Tukey double arcsine transformations [33]. Then, we determined whether the original rates should be transformed or not and which transformation method should be selected according to the testing results. In the current meta-analysis, arcsine-transformed proportions were used. Heterogeneity between studies was evaluated using Cochran’s chi-squared test (Cochran’s Q) and I^2^ values. When *P* was < 0.1 or I^2^ was < 50%, homogeneity between studies was assumed, and a fixed-effects model was adopted to calculate the pooled prevalence; conversely, a random-effects model was adopted. In this study, because of the existing significant heterogeneity, a random-effects model was adopted to calculate the estimates. To explore the possible sources of heterogeneity, sub-group analyses were conducted based on different categories: year of publication (1983 ~ 1989 vs 1990 ~ 1999 vs 2000 ~ 2004 vs 2005 ~ 2009 vs 2010 ~ 2015), geographic location (Central China vs South China vs North China vs East China vs Southwest vs Northwest vs Southwest vs Hong Kong/Taiwan), origin of sample (school vs general population), sample size (<1,000 vs 1,000 ~ 5,000 vs 5,001 ~ 10,000 vs > 10,000), clinical interview (yes vs no), diagnostic criteria (DSM vs CCMD vs ICD), source of information (“and rule” vs “or rule” vs clinicians vs parents vs teachers vs subjects vs unclear), gender of subjects (male vs female), age of subjects (children vs children and adolescents) and different subtypes (ADHD-I vs ADHD-HI vs ADHD-C). Publication bias of the studies was evaluated by testing for funnel plot asymmetry and conducting Egger’ s linear regression test. To test the robustness of this analysis, sensitivity analyses were conducted in studies with a low risk of bias versus the overall included studies. All analyses were performed using R3.1.2.

## Results

### Literature search

**Fig. 1:**
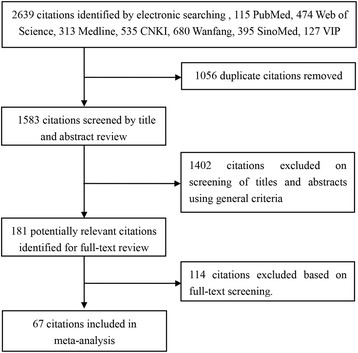
Flow diagram of included/excluded studies

In total, 2,639 studies were identified after an initial search. After removing duplicates and screening the titles and abstracts, 181 articles were potentially eligible and were reviewed in full text. After reading these articles carefully, 114 studies were excluded (47 duplicate publications, 25 without prevalence rates, 22 without a DSM/CCMD/ICD diagnosis, ten without diagnostic criteria and with a self-compiled questionnaire as the assessment tool, six with adults as the subjects, two retrospective studies, two with data that could not be extracted). Finally, a total of 67 studies were included in the meta-analysis (Fig. 1), and a full reference list is provided in Additional file 1.

### Characteristics of studies

The 67 studies included in the systematic review and meta-analysis were published between 1983 and 2015, with nearly 70% of the studies concentrated from 2005 to 2015. The sample sizes of the included studies ranged from 184 to 18,096, with a total of 275,502 people. The geographic locations included East China (20 studies), Central China (ten), South China (11), Southwest China (seven), North China (six), Northwest China (five), Northeast China (four) and Hong Kong/Taiwan (four). Most of the studies (64, 95.5%) were cross-sectional in design, and only three studies used a prospective cohort. With the exception of three studies based on the general population, the other 64 studies targeted school populations. More than half of the studies (39, 58.2%) applied a clinical interview, with only those screening positive in the first stage being interviewed. Nearly half of the studies (31, 46.3%) were only based on children, while the others targeted both children and adolescents. Regarding the diagnostic criteria, the DSM was adopted in 58 studies, including the DSM-IV (43), DSM-III-R (eight), DSM-III (six) and DSM-5 (one); the CCMD was used in eight studies, including the CCMD-3 (four), CCMD-2-R (three), and CCMD-2 (one); and the ICD (ICD-9) was used in only one study. The characteristics of each studies included in this meta-analysis are provided in Additional file 2.

Moreover, 55 studies described the prevalence rates between genders, only one study reported the rate among males, and 11 studies reported the total prevalence. Twenty-seven studies reported the prevalence rates of the different subtypes of ADHD.

### Assessment of risk of bias

Of all the included studies, 19.4% (13 studies) had a low risk of bias, 62.7% (42 studies) had a moderate risk and 17.9% (12 studies) had a high risk. None of the studies met all nine criteria. The overall selection bias was high, as none of the studies’ target population was a close representation of the national population regarding the prevalence of ADHD among children and adolescents, and the sampling frames were a true or close representation of the target population in only 25 studies. Only 4 studies (6%) collected information directly from children or adolescents. The details of the assessment of individual studies are shown in Additional file 3.

### Overall ADHD

**Fig. 2:**
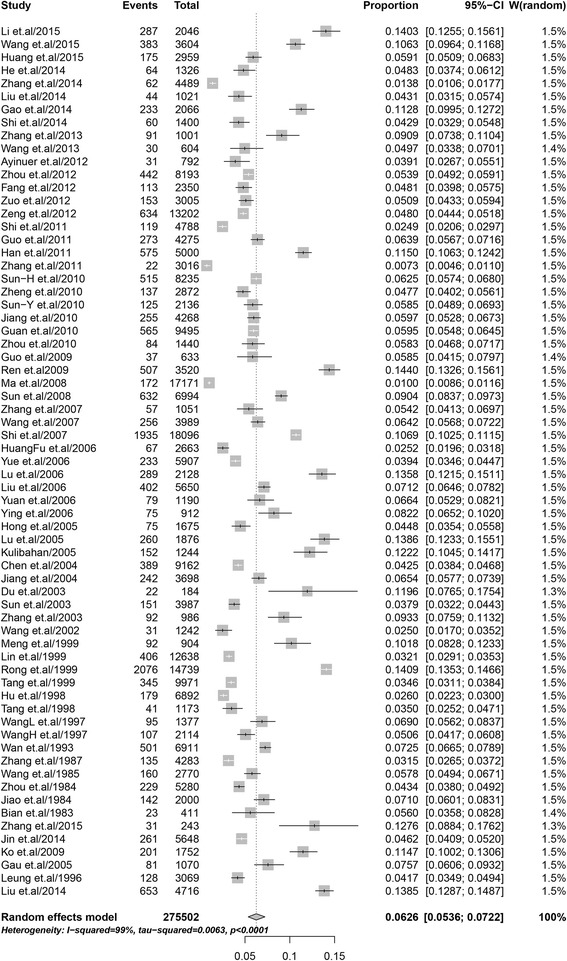
Forest plot of the prevalence of ADHD among children and adolescents in China

The point prevalence of ADHD reported in the included studies ranged from 0.73 to 14.40% with a pooled prevalence of 6.26% (95% CI: 5.36–7.22%) (Fig. 2). The analysis revealed significant heterogeneity between studies (I^2^ = 99.0%, *P* < 0.001).

It is worth noting the significant heterogeneity between the included studies. To explore the possible sources of heterogeneity, we performed subgroup analyses (Table 1). The variables “geographic location” (Q = 30.08, *P* < 0.001) and “source of information” (Q = 11.96, *P* = 0.035) partially explained the heterogeneity in this meta-analysis, while the variables “year of publication”, “origin of sample”, “sample size”, “clinician interview”, “diagnostic criteria” and “age of subjects” all failed to explain the source of heterogeneity (all *P* > 0.05).

**Table 1:** Prevalence of ADHD among children and adolescents in China: subgroup meta-analysis and analysis of heterogeneity

Characteristics	No.of studies	Event	n	Prevalence (%) (95% CI)	I^2^ (%)	*P* value for heterogeneity	Test for subgroup differences	
							Q	*P* value
Year of publication							3.10	0.541
2010 ~ 2015	28	6,417	104,190	6.02 (4.88, 7.72)	98.5	< 0.001		
2005 ~ 2009	18	5,510	77,521	7.51 (5.15, 10.27)	99.4	< 0.001		
2000 ~ 2004	6	927	19,259	5.62 (4.06, 7.41)	95.0	< 0.001		
1990 ~ 1999	10	3,970	59,788	5.63 (3.24, 8.63)	99.5	< 0.001		
1983 ~ 1989	5	689	14,744	5.05 (3.72, 6.57)	93.0	< 0.001		
Geographic location							30.08	< 0.001
Central China	10	2,165	37,611	6.16 (4.54, 8.02)	97.9	< 0.001		
East China	20	3,651	71,968	5.42 (4.54, 6.38)	96.6	< 0.001		
Hong Kong/Taiwan	4	1,063	10,607	8.90 (4.47, 14.64)	98.8	< 0.001		
North China	6	3,032	32,691	7.47 (3.52, 12.74)	99.5	< 0.001		
Northeast	4	1,474	30,542	6.33 (1.36, 14.56)	99.8	< 0.001		
Northwest	5	1,001	9,915	9.99 (8.28, 11.83)	88.2	< 0.001		
South China	11	4,310	60,569	6.82 (4.85, 9.11)	99.0	< 0.001		
Southwest	7	817	21,599	3.49 (1.75, 5.79)	98.4	< 0.001		
Origin of sample							1.83	0.176
School	64	16,575	254,711	6.37 (5.43, 7.38)	99	< 0.001		
General population	3	938	20,791	4.12 (1.78, 7.38)	98.8	< 0.001		
Sample size							5.73	0.125
< 1,000	9	433	5,669	7.65 (5.87, 9.64)	85.7	< 0.001		
1,000 ~ 5,000	41	7,164	105,649	6.35 (5.19, 7.61)	98.5	< 0.001		
5,001 ~ 10,000	12	4,693	88,338	5.21 (4.24, 6.28)	97.9	< 0.001		
> 10,000	5	5,223	75,846	5.83 (1.95. 11.62)	99.9	< 0.001		
Clinical interview							3.47	0.062
Yes	39	9,556	163,878	5.47 (4.61, 6.41)	98.4	< 0.001		
No	28	7,957	111,624	7.46 (5.60, 9.56)	99.4	< 0.001		
Diagnostic criteria							0.73	0.695
DSM^a^	58	15,964	247,772	6.20 (5.22, 6.68)	99.1	< 0.001		
CCMD^b^	8	1,389	24,960	6.72 (4.39, 9.51)	98	< 0.001		
ICD^c^	1	160	2,770	5.78 (4.94, 6.68)	-	-		
Source of information							11.96	0.035
“And rule”	3	2,406	25,618	6.00 (1.99, 9.51)	99.8	< 0.001		
Clinicians	39	9,556	163,878	5.47 (4.64, 6.42)	98.4	< 0.001		
Parents	16	3,508	60,196	7.29 (4.59, 8.35)	99.3	< 0.001		
Teachers	3	292	5,253	6.96 (4.12, 10.48)	93.4	< 0.001		
Subjects	3	1,083	11,748	9.43 (3.74, 17.35)	99.7	< 0.001		
Unclear	3	668	8,809	8.05 (6.82, 9.39)	63.9	0.063		
Age of subjects							1.05	0.306
Children	31	6,631	104,343	5.74 (4.48, 7.14)	98.8	< 0.001		
Children and adolescents	36	10,882	171,159	6.72 (5.46, 8.11)	99.2	< 0.001		
Gender of subjects							4.72	0.030
Male	56	10,913	132,904	8.17 (6.94, 9.50)	98.6	< 0.001		
Female	55	8,519	124,391	6.22 (5.07, 7.48)	98.7	< 0.001		
Different sub-types							30.7	0.000
ADHD-I	26	3,183	103,132	3.24 (2.52, 4.04)	97.9	< 0.001		
ADHD-HI	26	1,223	103,132	1.16 (0.87, 1.48)	95.1	< 0.001		
ADHD-C	26	1,952	103,132	1.71 (1.33, 2.13)	95.9	< 0.001		

^a^The DSM includes the DSM-5, DSM-IV, DSM-III-R and DSM-III; ^b^The CCMD includes the CCMD-3, CCMD-2-R and CCMD-2; ^c^The ICD refers to the ICD-9

Furthermore, multiple comparisons of the prevalence estimates of ADHD reported by different sources of information were performed. Data are shown in Additional file 4. With the exception for the prevalence estimates reported in studies with clinicians as the informant versus studies with an unclear informant (Q = 10.77, *P* = 0.001), no significant differences were found between any other subgroups (all *P* > 0.05).

In addition, the prevalence rates of ADHD based on gender and different criteria (DSM and CCMD) were compared. The summarized prevalence of male (8.17%, 95% CI: 6.94–9.50%) was significant higher than that of female (6.22%, 95% CI: 5.07–7.48%) (Table 1). In studies using the DSM, the pooled estimates of ADHD in studies applying the DSM-III, DSM-III-R, DSM-IV and DSM-5 were 4.27% (95% CI: 3.50–5.11%), 6.85% (95% CI: 4.21–10.06%), 6.36% (95% CI: 5.17–7.67%) and 5.91% (95% CI: 5.09–6.79%), respectively; the prevalence of ADHD based on the DSM-III was significantly lower than those based on the DSM-IV and DSM-5 (Q_DSM-III vs DSM-IV_ = 8.04, Q_DSM-III vs DSM-5_ = 7.54; all *P* < 0.05), while the differences between other DSM editions were not significant (all *P* > 0.05) (data not shown). In studies based on the CCMD, the pooled estimates of ADHD in studies applying the CCMD-2, CCMD-2-R and CCMD-3 were 3.50% (95% CI: 2.52–4.62%), 5.82% (95% CI: 2.77–9.91%) and 8.33% (95% CI: 6.67–10.15%), respectively; the prevalence of ADHD based on the CCMD-2 was significantly lower than that based on the CCMD-3 (Q = 23.26, *P* < 0.001), while the differences between the other editions of the CCMD were not significant (all *P* > 0.05) (data not shown).

### DSM-IV subtypes

As seen in Table 1, the pooled-prevalence estimates of ADHD-I, ADHD-HI and ADHD-C were 3.24% (95% CI: 2.52–4.04%), 1.16% (95% CI: 0.87–1.48%) and 1.71% (95% CI: 1.33–2.13%), respectively, with significant differences between them (Q = 30.7, *P* < 0.001). Further multiple comparisons were performed. The results showed that the pooled prevalence of ADHD-HI was significantly higher than that of ADHD-C (Q = 4.69, *P* = 0.030), and the pooled prevalence of ADHD-I was higher than the two other subtypes (Q_ADHD-I vs ADHD-HI_ = 30.69, Q_ADHD-I vs ADHD-C_ = 13.54, all *P* < 0.001) (data not shown).

### Publication bias and sensitivity analysis

**Fig. 3:**
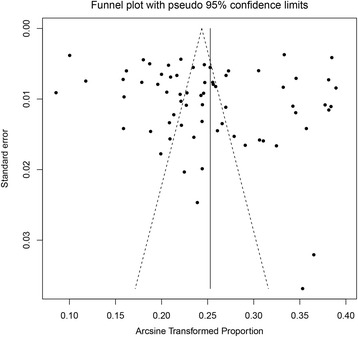
Funnel plots with 95% confidence limits of the prevalence of ADHD. The horizontal line represents the summary effect estimates, and the dotted lines are pseudo 95% CIs

Funnel plots and Egger’s test were combined to explore the potential publication bias in this meta-analysis. As shown in the funnel plot of the 67 included studies (Fig. 3), no evidence of publication bias was visually observed. Additionally, the t-score of Egger’s test was 0.800 (*P* = 0.427). Therefore, there was no evidence of significant publication bias in this meta-analysis.

Sensitivity analyses were conducted with the 13 studies that had a low risk of bias. The estimates of ADHD in these studies (6.82%, 95% CI: 4.33–9.82%) were slightly but not significantly higher than the overall pooled estimates (Q = 0.15, *P* = 0.703), indicating the robustness of this analysis.

## Discussion

There are currently no nationwide data on the prevalence of ADHD among children and adolescents in China. This systematic review and meta-analysis was performed to review the relevant observational studies conducted in the past 30 years in China, including 67 studies and more than 334,000 people. By reviewing studies within China, we included original studies reporting the prevalence of ADHD in eight regions of China. Although nearly half of the studies were conducted in East China and South China, the literature search showed that ADHD was a well-studied mental disorder. Therefore, it could be stated that this meta-analysis provided a more accurate value than the individual studies alone. The major findings are listed as follows: 1) the pooled prevalence rate of ADHD among children and adolescents in China was 6.26% (95% CI: 5.36–7.22%); 2) the prevalence of ADHD-I was the highest of the subtypes, followed by ADHD-HI and ADHD-C; and 3) the apparent inconsistency in prevalence rates was partially caused by the between-study differences in “geographic location” and “source of information”. In addition, the prevalence rates of ADHD did not change significantly between different time periods. The results of the sixth national census in our country showed that the number of people under 18 years old is approximately 367 million, suggesting that ADHD affects approximately 23 million children and adolescents in China.

The prevalence calculated in this meta-analysis was slightly inconsistent with a previous review in China (5.7%, 95% CI: 4.9–6.6%) [34]. This inconsistency might be explained by the increased number of included studies and the differences in the inclusion criteria. As almost all of the studies in China are published in Chinese or English, we included documents that had been published in Chinese and English by 2016 and used the DSM/ICD/CCMD as the diagnostic criteria. However, a previous meta-analysis [34] that included all of the 36 studies published before 2011, was conducted with a language restriction to Chinese and failed to distinguish whether these studies used recognized diagnostic criteria (DSM /ICD/CCMD). Nearly 1 in 5 of the studies simply identified patients by the results of screening scales [34]. Because of the limitation of scales in identifying patients [35], the findings of Tong et al. [34] could not well represent the true epidemic status of ADHD among children and adolescents. In addition, our estimate was similar to the outcome (7.2%, 95% CI: 6.7–7.8%) of a meta-analysis conducted by Thomas et al. [20]. Therefore, it could be concluded that the prevalence of ADHD in China is generally consistent with the worldwide prevalence.

In our study, the pooled prevalence in males was significantly higher than that in females, which was consistent with the results reported in studies conducted by Willcutt et al. [27] and Polanczyk et al. [30]. Evidence shows that ADHD is a disease of polygenic inheritance [36]. As girls have a higher genetic predisposition threshold than boys, girls show behavioural symptoms only when more relevant genes are involved [37]. In addition, the symptoms of hyperactivity and impulsivity are apparent in boys with ADHD, as well as the disruptive behaviours, such as aggression, disciplinary violations and other behaviours violating social norms, which are easily observed by parents and teachers and makes boys with ADHD more likely to be identified than girls with ADHD. Dissimilarly, girls with ADHD are less likely to have disruptive behaviour disorders and report higher prevalence of symptoms of inattention which are more covert than those of hyperactivity and impulsivity [38]. Moreover, in China, the misconception that men are superior to women is still prevalent in many people’s minds. Family expectations for boys are higher than those for girls, leading to the inappropriate education methods (such as doting or violence) that may increase the likelihood of psychological and behavioural problems in boys. In addition, ADHD-I was the most common subtype of ADHD in the samples in our study, followed by ADHD-C, and finally ADHD-HI, which was consistent with the results of a previous meta-analysis conducted by Willcutt et al. [27]. Furthermore, in Willcutt’ s study, the proportion of individuals with ADHD-C in samples ascertained by clinicians was higher than that of individuals with ADHD-I. This difference suggests that although more individuals in the overall population fit the diagnostic criteria for ADHD-I, individuals who meet the diagnostic criteria for ADHD-C may be more likely to be referred to a clinic [39]. However, due to a lack of sufficient statistical data, our study failed to provide support for this argument.

As with most psychiatric disorders, ADHD lacks a specific aetiology or change in pathology and has no unique features or laboratory index to assist its diagnosis. The diagnosis was based on a history of ADHD and the observation and description of specific behaviours and symptoms. However, due to a lack of objectivity, the results may be affected by observers’ subjective consciousness. To standardize the diagnosis of ADHD, researchers have employed one of the following existing diagnostic criteria over time: the DSM-III [40], DSM-III-R [41], DSM-IV [2], DSM-5 [42], ICD-9 [43] and ICD-10 [44]. Some differences exist between the key points of those diagnostic criteria that are not trivial and may account for part of the inconsistency in results and for a large component of the uncertainty about the characteristics of hyperactivity. In the DSM-III, the diagnosis is based on core symptoms including developmental inattentiveness, inappropriate overactivity and impulsiveness that occur before the age of seven and last for at least more than 6 months; all three symptoms are necessary for the diagnosis. In the ICD-9, the criteria for symptoms are not specified in the same detail as in the DSM-III and more emphasis is placed on symptoms of inattention and overactivity than on impulsiveness in practice. However, in the DSM-III-R, the criteria for this disorder were revised. Instead of requiring all three symptoms of inattentiveness, impulsiveness, and hyperactivity, the DSM-III-R requires individuals to meet eight symptom criteria from a list of 14 symptoms of hyperactivity, inattention, and impulsiveness. That is, if a child presents with any two of the three symptoms in the DSM-III, a diagnosis of ADHD would be recorded. Therefore, it can be deemed that the DSM-III-R is more inclusive than the DSM-III. In the DSM-IV and the ICD-10, the items in the list of symptom have increased from 14 to 18 while the symptom dimensions have changed to attention deficit and hyperactivity/impulsivity. The most important difference between the two criteria is the number of items required to indicate a diagnosis. The DSM-IV requires more than six of nine symptoms of attention deficit or hyperactivity/impulsivity to be met before a diagnosis of ADHD can be made; however, the ICD-10 requires more than six of the nine symptoms of attention deficit as well as those of hyperactivity/impulsivity to be met at the same time. Therefore, the diagnostic criteria of the ICD-10 are more rigorous than those of the DSM-IV. In the DSM-5, the age of onset of ADHD has been modified to 12 years of age. With the exception of the newly added descriptions of adults’ performances in each symptom criterion, the diagnostic criteria for ADHD in the DSM-5 are similar to those of the DSM-IV. In addition, compared to the other editions, the DSM-5 is able to diagnose both autism spectrum disorder (ASD) and ADHD simultaneously. The DSM and ICD are both widely used worldwide. However, because of the differences in concepts and cultures, the DSM and ICD are not entirely applicable to China. To address this discrepancy, the branch of the Chinese Medical Association (CMA) has formulated a series called the Chinese Classification of Mental Disorders (CCMD) (including the CCMD-2 [45], CCMD-2-R [46], and CCMD-3 [47]) based on the ICD. What differs is that the CCMD considers the influence of nationality, culture, age, gender, age of onset, parents’ cognition of ADHD and other factors on the diagnosis of ADHD, and the description of symptoms is more in line with conventional Chinese language; additionally, the CCMD requires fewer symptom items to be met for a diagnosis of ADHD to be made. Considering the CCMD-3 [47] as an example, in terms of its similarities to the ICD-10, the CCMD also includes a list of 18 symptoms items of attention deficit and hyperactivity/impulsivity, and if a child meets four of nine symptoms items for attention deficit as well as for hyperactivity/impulsivity, a diagnosis of ADHD can be made. In our country, the consistency between the CCMD-3 and a clinical diagnosis was found to be higher when compared with the DSM-IV or ICD-10 [48]. Therefore, the CCMD-3 may be more applicable than the DSM-IV and ICD-10 in China. However, there are very few studies on the application of the CCMD-3. Additionally, because of the limitations in international communication, the CCMD-3 has not yet been promoted in China. As previously reported, by December 31, 2004, 78.02% of the studies on ADHD among children and adolescents in China adopted the DSM diagnostic criteria (mainly the DSM-IV, 55.17%), followed by the CCMD (18.53%) and ICD (3.45%) [49]. Similarly, 86.57% of the studies included in our meta-analysis were based on the DSM, mainly the DSM-IV (64.20%), followed by the CCMD (11.94%) and ICD (1.5%, only one study). Unlike Polanczyk et al. [30], we did not find a significant difference in the pooled ADHD prevalence between the different diagnostic criteria. However, the estimates vary significantly between different editions, for both the DSM and CCMD. It is believed that since the release of the DSM-III, the reported prevalence of ADHD has gradually increased with each new edition [30, 50, 51]. Additionally, it was hypothesized in a meta-analysis that the estimates would increase significantly between studies according to their use of the DSM-III, DSM-III-R, and DSM-IV. However, this hypothesis was not strongly supported by the results [20]. In our study, although no linear trends were found, the pooled prevalence of ADHD in studies based on the DSM-III was lower than that of studies using the DSM-III-R, DSM-IV and DSM-5, and the differences between the DSM-III and DSM-IV as well as between the DSM-III and DSM-5 were statistically significant. Nevertheless, because of the imbalance in distribution of the included studies by edition, the results must be interpreted with caution.

It has been suggested that due to a certain degree of limitations and deficiencies, scales can only play a supporting rather than a definitive role in the clinical diagnosis of ADHD [52]. Furthermore, the prevalence of ADHD among children and adolescents might be overestimated or underestimated in epidemiological investigations that only use scales as the diagnostic method [52]. In addition, according to Willcutt et al. [27] and Polanczyk et al. [30], prevalence estimates are significantly lower when the informants are clinicians compared with when they are any other type of informant, which might help better establish population-based benchmarks for clinicians to consider. In our study, although the pooled estimates did not significantly differ between studies with or without a clinical interview, the estimates were lower when studies included a clinical interview for diagnose of ADHD, which supported the importance and necessity of clinical interviews to standardize the diagnosis of ADHD. Unlike the previous studies [27–30], we did not find any significant differences between the estimates reported in studies with children/adolescents, parents or teachers as the informants.

As reported in studies conducted by Tomas et al. [20] and Tong et al. [34], geographic location also contributed to the variation in the prevalence of ADHD, and the difference remained significant after controlling for the differences in diagnostic criteria. However, Willcutt et al. [27] and Polanczyk et al. [30] found that the differences between the prevalence estimates in countries or regions of the world lost significance when controlling for differences in the diagnostic algorithms used to define ADHD in children and adolescents. In this study, significant differences in prevalence were found between regions of China, suggesting that geographic differences may influence the prevalence of ADHD among children and adolescents in China. Studies have suggested that the differences between local social economies, cultures and educational standards may influence parenting styles, thereby affecting the personality and psychology of children [26, 53, 54]. As the estimate of heritability of ADHD is approximately 80% [55, 56], cultural and environmental factors also play a role in the aetiology of the disorder. In addition, a study that compared the consistency of results regarding children’ s behavioural problems evaluated by psychiatrists in China and the UK showed that individual cultural differences in the perception of behaviour and symptoms may result in the data inconsistencies observed in epidemiological studies; despite using uniform diagnostic criteria and symptom rating scales as diagnostic tools, the prevalence of ADHD remains inconsistent in regions with different cultures [57].

Although the present meta-analysis included 67 studies with a large sample size, there were still some limitations. First, there was clear heterogeneity between studies, which could partially be explained by “geographic location” and “source of information” according to the subgroup analysis. We hypothesize that other variables could affect the heterogeneity, such as investigation time, genetic and environmental factors, and family education methods. However, we could not obtain any information about these variables. The second limitation was that we included documents published in Chinese or in English, which may have led to the omission of a small proportion of studies. Thirdly, to ensure that the reported prevalence rates in each study were as homogeneous as possible, we used the most conservative diagnosis when extracting prevalence rates from the studies with more than one estimation. However, at present, there is a lack of definitive evidence supporting the accuracy and reliability of information from different sources; fortunately, this situation pertained to only six out of 67 studies. Finally, although the included studies covered 28 provinces and cities in China, a small proportion of the regions were not assessed, including Tibet, and the impact on the pooled prevalence was unclear.

## Conclusions

The prevalence of ADHD among children and adolescents in China is generally consistent with the worldwide prevalence and shows that ADHD affects quite a large number of people under 18 years of age. Despite the study limitations, this meta-analysis provides a valuable way of estimating the prevalence of ADHD among children and adolescents in China; the results can offer a suitable benchmark to assess the disease burden caused by ADHD as well as provide references for the optimization of health resource allocation and for the formulation of relevant health policies. However, a nationwide study is needed to provide more accurate estimations.

## Additional files

Additional file 1: “The list of 67 studies included in the meta-analysis”. (DOC 297 kb)
Additional file 2: “Characteristics of the included studies on the ADHD prevalence among children and adolescents in China”. (DOC 265 kb)
Additional file 3: “Risk of bias in individual studies”. (DOC 227 kb)
Additional file 4: “Multiple comparisons of the prevalence estimates of ADHD reported by different sources of information”. (DOC 27 kb)

## Abbreviations

ADHD: Attention deficit/hyperactivity disorder
ASD: Autism spectrum disorder
CCMD: Classification and Diagnostic Criteria of Mental Disorders in China
CMA: Chinese Medical Association
CNKI: China National Knowledge Infrastructure
DSM: Diagnostic and Statistical Manual of Mental Disorders
ICD: International Classification of Diseases
