# Supplementary material for: Prevalence of attention deficit/hyperactivity disorder among children and adolescents in China: a systematic review and meta-analysis
Source: BMC Psychiatry. 2017 Jan 19;17:32. doi: 10.1186/s12888-016-1187-9 (PMC5244567; doi:10.1186/s12888-016-1187-9)
Supplement: Additional file 1: — “The list of 67 studies included in the meta-analysis”. (DOC 297 kb) [file 12888_2016_1187_MOESM1_ESM.doc]

**Additional file 1**

The list of sixty-seven studies included in the meta-analysis

1. Zhang R, Huo X, Ho G, Chen X, Wang H, Wang T, et al. Attention-deficit/hyperactivity symptoms in preschool children from an e-waste recycling town: assessment by the parent report derived from DSM-IV. BMC Pediatr. 2015; 15:51.

2. Li Y, Huang M, Yu Q, Zhao B. Investigation of children and adolescents with attention deficit hyperactivity disorder combined with conduct disorder. Journal of International Psychiatry. 2015; 42:32-5.

3. Wang S, Ye X, Chen H, Ou G, Meng N, Mu J, et al. Prevalence of Attention Deficit Hyperacitivity Disorder and Ralated Factors among 6-13-Year-Old School Children in Lanzhou City. Practical Clinical Medicine. 2015; 16:90-5.

4. Huang Y. An epidemiological survey and clinical characteristics of ADHD in pupils in Shantou, Guangdong, China. Shantou University (Shantou); 2015. MA thesis.

5. Liu T, Yang P, Ko C, Yen J, Yen C. Association between ADHD symptoms and anxiety symptoms in Taiwanese adolescents. J Atten Disord. 2014; 18:447-55.

6. He M, Su Z, Peng R, Fan Y. Prevalence survey analysis of preschoolers obstructive sleep apnea syndrome and attention deficit hyperactivity disorder in liwan district of Guangzhou city. Chinese Community Doctors. 2014; 30:136-8.

7. Zhang C, Ai R, Deng B. ADHD among preschool students in Guiyang kindergartens. Chinese Journal of School Health. 2014; 35:60-1.

8. Gao Q. Study on the cognitive-behavior intervention of the ADHD children. Xinjiang Normal University(Ürümqi); 2014. MA thesis.

9. Shi L, Wang F. An epidemiological survey of attention deficit hyperactivity disorder in children aged 4 to 9 years in Leshan City, Sichuan Province. Maternal and Child Health Care of China. 2014; 29:1734-5.

10. Liu F, Liao L, Jiang Z. Analysis of Related Factors of Attention Deficit Hyperactivity Disorder among Children between 6 and 12 Years old in LiuZhou. Contemporary Medicine Forum. 2014; 12:179-81.

11. Jin W, Du Y, Zhong X, David C. Prevalence and contributing factors to attention deficit hyperactivity disorder: a study of five- to fifteen-year-old children in Zhabei District, Shanghai. Asia Pac Psychiatry. 2014; 6:397-404.

12. Zhang H, Lu G. Preliminary Exploration of Children attention deficit hyperactivity disorder and Sleep Disorders. China Practical Medicine. 2013; 08:43-5.

13. Wang L, Liu J, Wei Y. Relationship between attention deficit hyperactivity disorder in preschool children and child neglect. Chinese Journal of Woman and Child Health Research. 2013; 24:144-6.

14. Ayinuer W, Jin W, Du Y, Zhong X, David C. Study on Prevalence and Influence Factors of ADHD among Children in Preschool. China Medical Devices. 2012; 27:17-20.

15. Zhou K, Gao M, Yang C, Zhang J, Chen Y, Song J, et al. An epidemiological survey of attention deficit hyperactivity disorder in school- age children in Shenzhen. Chinese Journal of Contemporary Pediatrics. 2012; 14:689-92.

16. Fang M, Yang Y, Wang X, Tang Y, Zhuang X. Diagnosis of Puerile Attention Deficit Hyperactivity Disorder in Xiehang City Liangshan Yi Autonomous Prefecture，Sichuan Province. Chinese Journal of Obstetrics & Gynecology and Pediatrics. 2012; 08:721-4.

17. Zou M, Shu Q, Ai L, Dong J, Luo W, Wei X, et al. Investigation and analysis of ADHD and obstructive sleep apnea hypopnea syndrome in primary school students in Xiaogan. Journal of Clinical Pulmonary Medicine. 2012; 17:1312-3.

18. Zeng M. Epidemiological survey of attention deficit hyperactivity disorder in children aged 7-11 years in Yangjiang. Health. 2012; 08:443.

19. Shi Q, Zhou S, Wu Z. 7-10 years old children in different subtypes of ADHD behavior and comorbid problems. Psychological Science. 2011; 34:1516-9.

20. Guo H, Chen G. Investigation on the related factors of attention deficit hyperactivity disorder among children aged 6 to 16 years in Binzhou. Shandong Medical Journal. 2011; 51:77-8.

21. Han L, Han Y, Huang G, Li J. Epidemiological survey of attention deficit hyperactivity disorder among children and adolescents in Liaoyang city of Liaoning province and its correlation with various factors. Medical Journal of Chinese People's Health. 2011; 23:883-7.

22. Sun H. Study on related factors of attention deficit hyperactivity disorder in children aged 4~16 years old. Maternal and Child Health Care of China. 2010; 25:2519-21.

23. Zheng W. Epidemiological survey and evaluation of the effect of ADHD in children with attention deficit hyperactivity disorder. Chinese Journal of Child Health Care. 2010; 18:329-30.

24. Sun Y, Sun D. Univariate analysis on family environment to attention deficit hyperactivity disorder among children aged 4-5 years old. Chinese Journal of Child Health Care. 2010; 18:994-6.

25. Jiang H, Yu J, Yu T, Wang Q, Wang H, Wang J. An epidemiological survey on ADHD in school age children of Weihai. Journal of Psychiatry. 2010; 23:116-8.

26. Guan BQ. Psychological abuse in children with attention deficit hyperactivity disorder: an epidemiological survey. Central South University (Changsha); 2010. MA thesis.

27. Zhou X, Wang Y, Hu W. Epidemiological investigation on children with ADHD in Qujiang District. Zhejiang Medical Journal. 2010; 32:612-3.

28. Zhu Y, Zhang B, Deng B. Epidemiological Investigation of ADHD Among Elementary and Middle School Students in Guiyang. Chinese Journal of School Health. 2010; 31:62-4.

29. Ko C, Yen J, Chen C, Yeh Y, Yen C. Predictive values of psychiatric symptoms for internet addiction in adolescents: a 2-year prospective study. Arch Pediatr Adolesc Med. 2009; 163:937-43.

30. Guo M, Huang G, Zhang F, Yu F, Chao X, Li M, et al. Study and Logistic Analysis on Attention Deficit Hyperactivity Disorder Among 633 Puplis. Modern Preventive Medicine. 2009; 36:3651-3.

31. Ren L, Liu G, Tong W, Wang W, Deng S, Qian G. Cohort Study on the Relationship Between Parental Rearing Pattems and ADHD Among Preschool Children. Chinese Journal of School Health. 2009; 30:429-31.

32. Ma C. An epidemiological survey of Attention-Deficit Hyperactivity Disorder (ADHD) among 6 to 17-year-old students in Jilin province. Jilin University (Changchun); 2008. MA thesis.

33. Sun D, Yang Y, Song Y, Yu S. Epidemiological investigation of ADHD in Mudanjiang. Sichuan Mental Health. 2008; 21:164-6.

34. Zhang W, Liu X, Gu Q, Liao R, Ran L. An Epidemiological Investigation of ADHD in Six Cities. Chinese Journal of Clinical Psychology. 2007; 15:23-5.

35. Wang X, Yang M, Yang H, Jiang R, Liu Y, Bai S, et al. The Prevalence of ADHD Among Pupils in Xiamen. Chinese Journal of School Health. 2007; 28:995-6.

36. Shi S, Su G, Deng X, Tan H, Cai J, Shi Q, et al. Unfold a screen of Attention-Deficit Hyperactivity Disorder using ADHD software. Modern Hospital. 2007; 07:138-9.

37. Huangfu Z. Epidemiological study and family factors of children with Attention-Deficit Hyperactivity Disorder in Foshan. China Journal of Modern Medicine. 2006; 16:149-50.

38. Yue Y, Li Y, Li Y, Yue L, Lu A. Prevalence of Attention-Deficit Hyperactivity Disorder in children in Huaibei City. Chinese Journal of School Health. 2006; 27:454.

39. Lv L, Shi Q, Tao F, Yin P, Yin S. Epidemiological Stdy on Subtypes of ADHD of Children Aged 4-16 Years Old in Wuhan. Chinese Mental Health Journal. 2006; 20:221-5.

40. Liu H, Zheng F, Shi S, Shi M, Ni L, Zhou J, et al. Study on the relationship between the Attention -Deficit Hyperactivity Disorder and serum elements in 4 ethnic groups in Yunnan Province. Chinese Journal of School Doctor. 2006; 20:349-51.

41. Yuan X. Epidemiological survey of Attention-Deficit Hyperactivity Disorder in primary and middle school students in Changsha City. Central South University (Changsha); 2006. MA thesis.

42. Ying W, Zhu X. Investigation on the prevalence of ADHD in a primary school in Heze City. Preventive Medicine Tribune. 2006; 12:762.

43. Gau S, Chong M, Chen T, Cheng A. A 3-year panel study of mental disorders among adolescents in Taiwan. Am J Psychiatry. 2005; 162:1344-50.

44. Hong J. Family risk factors of attention deficit hyperactivity disorder in children. Chinese Journal of Clinical Rehabilitation. 2005; 09:64-6.

45. Lu H. Epidemiological Study of Attention-Deficit Hyperactivity Disorder Among School-age Children in Suzhou. Soochow University (Suzhou); 2005. MA thesis.

46. Kulibahan, Li D, Ye E, Ruo M, Panggejiapu. Investigation of attention deficit hyperactivity disorder among the ethnic Han and Kazakh students from Kuntum City of Xinjiang. Chinese Journal of Contemporary Pediatrics. 2005; 07:366-8.

47. Chen S, Zeng F, Tang H, Yang G, Chen Y, Li Q. Incidence and Related Factors of ADHD in Children of Guilin City. Chinese Journal of Clinical Psychology. 2004; 12:386-7.

48. Jiang L. Epidemiological Survey of Attention-deficient Hyperactivity Disorder and Related Factors in Zhenjiang Municipal Districts. The Second Military Medical University (Shanghai); 2004. MA thesis..

49. Du D, Li W, Chen J. Epidemiological analysis of children with ADHD in urban area of Guangzhou. Guangdong Medical Journal. 2003; 24:299-300.

50. Zhang J, Yang L, Chen Y, Huang Y. Investigation of children attention deficit hyperactivity disorder at three school in Zhundong. Chinese General Practice. 2003; 06:746-7.

51. Sun X, Pang Q, Zhao G, Zheng Y, Peng W, Cheng Y, et al. Epidemiologic Study of ADHD in Zibo City of Shandong Province. Chinese Mental Health Journal. 2003; 17:453.

52. Wang M, Luo S, Jin L, Wang C, Lin J. The analysis of behavior problems in preschool children with attention def icit -hyperactivity disorder. Chinese Journal of Behavioral Medical Science. 2002; 11:80-1.

53. Meng L, Zhao Z, Zheng F, Jiang Y, Huo K. Epidemiological study of attention deficit hyperactivity disorder of the children in Jiaozuo. The Henan medical researc. 1999; 08:364-5.

54. Lin Y, Zhuang H, Zheng Y. The Investigation and Research for Hyperactivity of School Age Children in Putian County. Fujian Medical Journal. 1999; 21:105-7.

55. Rong A, Zhang M. Investigation on ADHD among Urban and Rural Children of School Age Using Conners Questions and Answers Paper. Chinese Journal of School Health. 1999; 20:107-8.

56. Tang W, Ding Z. Prevalence of mild brain dysfunction in primary school students in Tianjin City. Chinese Mental Health Journal. 1999; 13:364.

57. Hu Y, Wu H, Yu J. Subtyping and inquiring for etiology of children ADHD aged 6 to 12 years. Chinese Journal of School Doctor. 1998; 12:321-4.

58. Tang J, Tu J, Cao H, Yang L, Que X. Investigation and analysis of ADHD in primary and secondary schools and kindergartens in Changsha City. Hunan Medical Journal. 1998; 15:185-6.

59. Wang L, Ying C, Li D, Yang Z, Peng T, Wei Y, et al. A epidemiological survey on childhood hyperkinetic syndrome among aged 7~10 in Dong Li Di strict of Harbin. Chinese Journal of Behavioral Medical Science. 1997; 06:46-8.

60. Wang H, Zhang H. Epidemiological survey of children with attention deficit hyperactivity disorder in Taiyuan City. Chinese Mental Health Journal. 1997; 11:47.

61. Leung P, Luk S, Ho T, Taylor E, Mak F, Bacon-Shone J. The diagnosis and prevalence of hyperactivity in Chinese schoolboys. Br J Psychiatry. 1996; 168:486-96.

62. Wan G, Yang Z. Epidemiological study of attention deficit hyperacticity disorder of 7-16 years old children in Hunan province. Chinese Journal of Psychiatry. 1993; 26:298-301.

63. Zhang M, Li Q, Xu J, Hu Y, Xi Y. Investigation report of mild brain dysfunction in urban and rural children in Leshan. Sichuan Medical Journal. 1987; 08:240-1.

64. Wang Y, Shen Y, Yang X. MBD investigation report of six primary schools in different areas of Beijing City. Chinese Journal of Nervous and Mental Diseases. 1985; 11:274-6.

65. Zhou M. Investigation report on children with ADHD in five primary schools in Nanchang City. Chinese Journal of Nervous and Mental Diseases. 1984:109-11.

66. Jiao F. Investigation on Incidence of MBD in 2,000 School-age Children. Journal of Xi' an Jiaotong University(Medical Sciences). 1984; 05:61-3.

67. Bian X, Xiang T, Dai X, Wang Z, Xie Z, Ma F, et al. Investigation on children with hyperactivity syndrome. Journal of North Sichuan Medical College. 1983:39-42.
