# Supplementary material for: Prevalence of attention deficit/hyperactivity disorder among children and adolescents in China: a systematic review and meta-analysis
Source: BMC Psychiatry. 2017 Jan 19;17:32. doi: 10.1186/s12888-016-1187-9 (PMC5244567; doi:10.1186/s12888-016-1187-9)
Supplement: Additional file 2: — “Characteristics of the included studies on the ADHD prevalence among children and adolescents in China”. (DOC 265 kb) [file 12888_2016_1187_MOESM2_ESM.doc]

**Additional file 2:**

Characteristics of the included studies on the ADHD prevalence among children and adolescents in China.

| First author | Year of publication | Geographic location | Origin of sample | Study design | Subjects | Age range or mean age (in  years) | Source of information | Assessment tools | Clinical interview | Diagnostic criteria | Sample size | Prevalence  estimate  (%) |
| --- | --- | --- | --- | --- | --- | --- | --- | --- | --- | --- | --- | --- |
| Li et.al | 2015 | North China | School | Cross- sectional | Children and adolescents | 4~16 | Parents | ADHD symptom questionnaire, CPRS | No | DSM-IV | 2046 | 14.03% |
| Wang et.al | 2015 | Northwest | School | Cross- sectional | Children and adolescents | 6~13 | Clinicians | CPRS | Yes | DSM-IV | 3604 | 10.63% |
| Huang et.al/ | 2015 | South China | School | Cross- sectional | Chlidren | 7~12 | Clinicians | ASQ, PRS, CTRS, SNAP-IV | Yes | DSM-5 | 2959 | 5.91% |
| He et.al | 2014 | South China | School | Cross- sectional | Chlidren | 3~6 | Clinicians | CPRS, CTRS | Yes | DSM-IV | 1326 | 4.83% |
| Zhang et.al | 2014 | Southwest | School | Cross- sectional | Chlidren | 3~6 | Clinicians | DSM-IV diagnostic scale | Yes | DSM-IV | 4489 | 1.38% |
| Liu et.al | 2014 | South China | School | Cross- sectional | Chlidren | 6~12 | Clinicians | SNAP-IV | Yes | DSM-IV | 1021 | 4.31% |
| Gao et.al | 2014 | Northwest | School | Cross- sectional | Children and adolescents | 6~15 | Parents | DSM-IV diagnostic scale | No | DSM-IV | 2066 | 11.28% |
| Shi et.al | 2014 | Southwest | School | Cross- sectional | Chlidren | 4~9 | Parents | ADHD symptom questionnaire | No | DSM-IV | 1400 | 4.29% |
| Zhang et.al | 2013 | Northwest | School | Cross- sectional | Children and adolescents | 5~13 | Clinicians | CTRS | Yes | DSM-IV | 1001 | 9.09% |
| Wang et.al | 2013 | East China | School | Cross- sectional | Chlidren | 3~6 | Clinicians | CPRS | Yes | DSM-IV | 604 | 4.97% |
| Ayinuer et.al | 2012 | East China | School | Cross- sectional | Chlidren | 3~6 | Clinicians | ADHD symptom questionnaire | Yes | DSM-IV | 792 | 3.91% |
| Zhou et.al | 2012 | South China | School | Cross- sectional | Children and adolescents | 7~13 | Clinicians | CPRS, CTRS | Yes | DSM-IV | 8193 | 5.39% |
| Fang et.al | 2012 | Southwest | School | Cross- sectional | Children and adolescents | 6~16 | Clinicians | ADHD symptom questionnaire | Yes | DSM-IV | 2350 | 4.81% |
| Zuo et.al | 2012 | Central China | School | Cross- sectional | Children and adolescents | 6~14 | Clinicians | CPRS, CTRS | Yes | DSM-IV | 3005 | 5.09% |
| Zeng et.al | 2012 | South China | School | Cross- sectional | Chlidren | 7~11 | Clinicians | Self-design questionnaires | Yes | DSM-III-R | 13202 | 4.80% |
| Shi et.al | 2011 | East China | School | Cross- sectional | Chlidren | 7~10 | Clinicians | SDQ | Yes | DSM-IV | 4788 | 2.49% |
| Guo et.al | 2011 | East China | School | Cross- sectional | Children and adolescents | 6~16 | Parents | ADHD symptom questionnaire, CBCL, CPRS | No | DSM-IV | 4275 | 6.39% |
| Han et.al | 2011 | Northeast | School | Cross- sectional | Children and adolescents | 7~16 | Clinicians | ADHD symptom questionnaire, CBCL, CTRS | Yes | DSM-IV | 5000 | 11.50% |
| Zhu et.al | 2010 | Southwest | School | Cross- sectional | Children and adolescents | 7~16 | Clinicians | CPRS | Yes | DSM-IV | 3016 | 0.73% |
| Sun et.al | 2010 | East China | School | Cross- sectional | Children and adolescents | 4~16 | Clinicians | ADHD symptom questionnaire | Yes | DSM-IV | 8235 | 6.25% |
| Zheng et.al | 2010 | East China | School | Cross- sectional | Chlidren | 8~12 | Clinicians | ASQ | Yes | DSM-IV | 2872 | 4.77% |
| Sun et.al | 2010 | East China | School | Cross- sectional | Chlidren | 4~5 | Clinicians | ADHD symptom questionnaire | Yes | DSM-IV | 2136 | 5.85% |
| Jiang et.al | 2010 | East China | School | Cross- sectional | Children and adolescents | 6~16 | Parents | ADHD symptom questionnaire | No | DSM-IV | 4268 | 5.97% |
| Guan et.al | 2010 | Central China | School | Cross- sectional | Children and adolescents | 5~17 | Clinicians | Self-design questionnaires | Yes | DSM-IV | 9495 | 5.95% |
| Zhou et.al | 2010 | East China | School | Cross- sectional | Children and adolescents | 6~14 | Clinicians | CPRS | Yes | CCMD-2-R | 1440 | 5.83% |
| Guo et.al | 2009 | Central China | School | Cross- sectional | Chlidren | 9~11 | Clinicians | CPRS, CTRS | Yes | CCMD-3 | 633 | 5.85% |
| Ren et.al | 2009 | South China | School | Prospective cohort | Chlidren | 5~6 | Parents | Self-design questionnaires | No | DSM-IV | 3520 | 14.40% |
| Ma et.al | 2008 | Northeast | School | Cross- sectional | Children and adolescents | 6~17 | Parents | CPRS, CTRS | No | DSM-IV | 17171 | 1.00% |
| Sun et.al | 2008 | Northeast | School | Cross- sectional | Children and adolescents | 6~14 | Parents | CPRS | No | CCMD-3 | 6994 | 9.04% |
| Zhang et.al | 2007 | North China | School | Cross- sectional | Children and adolescents | 6~14 | Parents | DSM-IV diagnostic scale, Self-design questionnaires | No | DSM-IV | 1051 | 5.42% |
| Wang et.al | 2007 | East China | School | Cross- sectional | Chlidren | 7~11 | Clinicians | CPRS | Yes | DSM-IV | 3989 | 6.42% |
| Shi et.al | 2007 | South China | General population | Cross- sectional | Children and adolescents | 3~14 | Clinicians | CPRS, CTRS | Yes | DSM-IV | 18096 | 10.69% |
| HuangFu et.al | 2006 | South China | School | Cross- sectional | Children and adolescents | 9.98±1.56 | Parents | ADHD symptom questionnaire | No | DSM-IV | 2663 | 2.52% |
| Yun et.al | 2006 | East China | School | Cross- sectional | Chlidren | 6~12 | Parents | ASQ, CPRS, CBCL | No | DSM-IV | 5907 | 3.94% |
| Lu et.al | 2006 | Central China | School | Cross- sectional | Children and adolescents | 4~16 | Clinicians | ADHD symptom questionnaire, CPRS, CTRS | Yes | DSM-IV | 2128 | 13.58% |
| Liu et.al | 2006 | Southwest | School | Cross- sectional | Children and adolescents | 6~14 | Clinicians | DSM-IV diagnostic scale, CPRS | Yes | DSM-IV | 5650 | 7.12% |
| Yuan et.al | 2006 | Central China | School | Cross- sectional | Children and adolescents | 5~17 | Clinicians | Self-design questionnaires, CBCL | Yes | DSM-IV | 1190 | 6.64% |
| Ying et.al | 2006 | East China | School | Cross- sectional | Chlidren | 6~13 | unclear | CCMD-3 diagnostic scale | No | CCMD-3 | 912 | 8.22% |
| Hong et.al | 2005 | East China | School | Cross- sectional | Chlidren | 6~14 | Clinicians | Self-design questionnaires | Yes | DSM-III-R | 1675 | 4.48% |
| Lu et.al | 2005 | East China | School | Cross- sectional | Chlidren | 6~13 | Parents | DSM-IV diagnostic scale, CPRS | No | DSM-IV | 1876 | 13.86% |
| Kulibahan | 2005 | Northwest | School | Cross- sectional | Children and adolescents | 8~16 | Clinicians | CPRS, CTRS | Yes | DSM-IV | 1244 | 12.22% |
| Chen et.al | 2004 | South China | School | Cross- sectional | Chlidren | 5~12 | Clinicians | CPRS, CTRS, DSM-IV diagnostic scale | Yes | DSM-IV | 9162 | 4.25% |
| Jiang et.al | 2004 | East China | School | Cross- sectional | Chlidren | 7~15 | Parents | ADHD symptom questionnaire | No | DSM-IV | 3698 | 6.54% |
| Du et.al | 2003 | South China | School | Cross- sectional | Children and adolescents | 5~15 | Teachers | CTRS | No | CCMD-3 | 184 | 11.96% |
| Sun et.al | 2003 | East China | School | Cross- sectional | Chlidren | 4~12 | And rule | CPRS, CTRS | No | DSM-III | 3987 | 3.79% |
| Zhang et.al | 2003 | East China | School | Cross- sectional | Chlidren | 7~12 | unclear | ASQ | No | CCMD-2-R | 986 | 9.33% |
| Wang et.al | 2002 | East China | General population | Cross- sectional | Chlidren | 3~6 | Clinicians | CBCL | Yes | DSM-IV | 1242 | 2.50% |
| Meng et.al | 1999 | Central China | School | Cross- sectional | Chlidren | 4~12 | Parents | Self-design questionnaires | No | DSM-III-R | 904 | 10.18% |
| Lin et.al | 1999 | East China | General population | Cross- sectional | Children and adolescents | 7~14 | Clinicians | ASQ | Yes | CCMD-2-R | 12638 | 3.21% |
| Rong et.al | 1999 | North China | General population | Cross- sectional | Chlidren | 7~12 | And rule | CPRS, CTRS | No | DSM-III-R | 14739 | 14.09% |
| Tang et.al | 1999 | North China | School | Cross- sectional | Children and adolescents | 6~13 | Clinicians | DSM-III diagnostic scale | Yes | DSM-III | 9971 | 3.46% |
| Hu et.al | 1998 | Central China | School | Cross- sectional | Chlidren | 6~12 | And rule | Self-design questionnaires | No | DSM-IV | 6892 | 2.60% |
| Tang et.al | 1998 | Central China | School | Cross- sectional | Chlidren | 4~14 | Clinicians | Self-design questionnaires | Yes | CCMD-2 | 1173 | 3.50% |
| WangL et.al | 1997 | Northeast | General population | Cross- sectional | Chlidren | 7~10 | Clinicians | Self-design questionnaires | Yes | DSM-III | 1377 | 6.90% |
| WangH et.al | 1997 | North China | School | Cross- sectional | Children and adolescents | 6~13 | Parents | DSM-III-R diagnostic scale | No | DSM-III-R | 2114 | 5.06% |
| Wan et.al | 1993 | Central China | General population | Cross- sectional | Children and adolescents | 7~16 | unclear | Self-design questionnaires | No | DSM-III-R | 6911 | 7.25% |
| Zhang et.al | 1987 | Southwest | School | Cross- sectional | Children and adolescents | 6~13 | Clinicians | Self-design questionnaires, CTRS | Yes | DSM-III | 4283 | 3.15% |
| Wang et.al | 1985 | North China | School | Cross- sectional | Chlidren | 7~12 | Clinicians | Self-design questionnaires | Yes | ICD-9 | 2770 | 5.78% |
| Zhou et.al | 1984 | Central China | School | Cross- sectional | Children and adolescents | 6~15 | Subjects | DSM-III diagnostic scale | No | DSM-III | 5280 | 4.34% |
| Jiao et.al | 1984 | Northwest | School | Cross- sectional | Children and adolescents | 7~15 | Teachers | DSM-III-R diagnostic scale | No | DSM-III-R | 2000 | 7.10% |
| Bian et.al | 1983 | Southwest | School | Cross- sectional | Children and adolescents | 6~13 | Clinicians | Self-design questionnaires | Yes | DSM-III | 411 | 5.60% |
| Zhang et.al | 2015 | South China | School | Cross- sectional | Chlidren | 3~7 | Parents | CPRS | No | DSM-IV | 243 | 12.76% |
| Jin et.al | 2014 | East China | School | Cross- sectional | Children and adolescents | 5~15 | Clinicians | ADHD symptom questionnaire | Yes | DSM-IV | 5648 | 4.62% |
| Ko et.al | 2009 | Hong Kong/Taiwan | School | Prospective cohort | Children and adolescents | 12~17 | Subjects | ADHDS | No | DSM-IV | 1752 | 11.47% |
| Gau et.al | 2005 | Hong Kong/Taiwan | School | Prospective cohort | Children and adolescents | 13~15 | Clinicians | K-SADS-E. CBCL | Yes | DSM-IV | 1070 | 7.57% |
| Leung et.al | 1996 | Hong Kong/Taiwan | School | Cross- sectional | Chlidren | 7~8 | Teachers | PACS, CTRS, Rutter's Teacher Questionnaire and Parent Questionnaire | No | DSM-III-R | 3069 | 4.17% |
| Liu et.al | 2014 | Hong Kong/Taiwan | School | Cross- sectional | Children and adolescents | 12~17 | Subjects | ADHDS | No | DSM-IV | 4716 | 13.85% |

CPRQ: Conners’ Parent Rating Scale; ASQ: Conners' Abbreviated Symptom Questionnaires; CTRS: Conners’ Teacher Rating Scale; DSM, Diagnostic and Statistical Manual of Mental Disorders; SNAP-IV: Swanson, Nolan, and Pelham Rating Scale, Version IV, Parent form and Teacher form; SDQ: Strengths and Difficulties Questionnaires; CBCL: Child Behavior Checklist; K-SADS-E: Kiddy Schedule for affective diseases and Schizophrenia (epidemiological version); ADHDS: ADHD Self-Rated Scale; PACS: Parental Account of Childhood Symptoms.
