# Supplementary material for: Prevalence of attention deficit/hyperactivity disorder among children and adolescents in China: a systematic review and meta-analysis
Source: BMC Psychiatry. 2017 Jan 19;17:32. doi: 10.1186/s12888-016-1187-9 (PMC5244567; doi:10.1186/s12888-016-1187-9)
Supplement: Additional file 3: — “Risk of bias in individual studies”. (DOC 227 kb) [file 12888_2016_1187_MOESM3_ESM.doc]

**Additional file 3**

**Risk of bias in individual studies**

| study | Q1 | Q2 | Q3 | Q4 | Q5 | Q7 | Q8 | Q9 | Q10 | Total number of "L" | Category |
| --- | --- | --- | --- | --- | --- | --- | --- | --- | --- | --- | --- |
| Li et.al/2015 | - | - | L | L | - | L | L | L | L | 6 | Moderate RoBs |
| Wang et.al/2015 | - | L | L | L | - | L | L | L | L | 7 | Low RoBs |
| Huang et.al/2015 | - | L | L | - | - | L | L | L | L | 6 | Moderate RoBs |
| He et.al/2014 | - | - | L | L | - | L | L | L | L | 6 | Moderate RoBs |
| Zhang et.al/2014 | - | L | L | - | - | L | L | L | L | 6 | Moderate RoBs |
| Liu et.al/2014 | - | L | L | - | - | L | L | L | L | 6 | Moderate RoBs |
| Gao et.al/2014 | - | - | - | L | - | L | L | L | L | 5 | Moderate RoBs |
| Shi et.al/2014 | - | - | L | - | - | L | L | L | L | 5 | Moderate RoBs |
| Zhang et.al/2013 | - | - | L | L | - | L | L | L | L | 6 | Moderate RoBs |
| Wang et.al/2013 | - | - | L | - | - | L | L | L | L | 5 | Moderate RoBs |
| Ayinuer et.al/2012 | - | - | L | - | - | L | L | L | L | 5 | Moderate RoBs |
| Zhou et.al/2012 | - | L | L | L | - | L | L | L | L | 7 | Low RoBs |
| Fang et.al/2012 | - | L | L | L | - | L | L | L | L | 7 | Low RoBs |
| Zuo et.al/2012 | - | - | - | L | - | L | L | L | L | 5 | Moderate RoBs |
| Zeng et.al/2012 | - | - | - | - | - | - | L | L | L | 3 | High RoBs |
| Shi et.al/2011 | - | - | - | - | - | L | L | L | L | 4 | High RoBs |
| Guo et.al/2011 | - | - | L | L | - | L | L | L | L | 6 | Moderate RoBs |
| Han et.al/2011 | - | - | L | L | - | L | L | L | L | 6 | Moderate RoBs |
| Zhu et.al/2010 | - | L | L | - | - | L | L | L | L | 6 | Moderate RoBs |
| Sun-H et.al/2010 | - | - | L | L | - | - | L | L | L | 5 | Moderate RoBs |
| Zheng et.al/2010 | - | - | - | L | - | L | L | L | L | 5 | Moderate RoBs |
| Sun-Y et.al/2010 | - | - | L | - | - | - | L | L | L | 4 | High RoBs |
| Jiang et.al/2010 | - | - | L | L | - | L | L | L | L | 6 | Moderate RoBs |
| Guan et.al/2010 | - | - | L | L | - | L | L | L | L | 6 | Moderate RoBs |
| Zhou et.al/2010 | - | L | L | L | - | L | L | L | L | 7 | Low RoBs |
| Guo et.al/2009 | - | - | - | L | - | L | L | L | L | 5 | Moderate RoBs |
| Ren et.al2009 | - | - | L | L | - | - | L | L | L | 5 | Moderate RoBs |
| Ma et.al/2008 | - | L | L | L | - | L | L | L | L | 7 | Low RoBs |
| Sun et.al/2008 | - | L | L | L | - | L | L | L | L | 7 | Low RoBs |
| Zhang et.al/2007 | - | - | L | L | - | L | L | L | L | 6 | Moderate RoBs |
| Wang et.al/2007 | - | L | L | L | - | L | L | L | L | 7 | Low RoBs |
| Shi et.al/2007 | - | - | L | - | - | L | L | L | L | 5 | Moderate RoBs |
| HuangFu et.al/2006 | - | - | L | L | - | - | L | L | L | 5 | Moderate RoBs |
| Yue et.al/2006 | - | - | L | L | - | L | L | L | L | 6 | Moderate RoBs |
| Lu et.al/2006 | - | - | L | L | - | L | L | L | L | 6 | Moderate RoBs |
| Liu et.al/2006 | - | - | - | - | - | L | L | L | L | 4 | High RoBs |
| Yuan et.al/2006 | - | L | L | L | - | L | L | L | L | 7 | Low RoBs |
| Ying et.al/2006 | - | L | - | - | - | - | L | L | L | 4 | High RoBs |
| Hong et.al/2005 | - | - | - | L | - | L | L | L | L | 5 | Moderate RoBs |
| Lu et.al/2005 | - | - | L | L | - | L | L | L | L | 6 | Moderate RoBs |
| Kulibahan/2005 | - | L | L | L | - | L | L | L | L | 7 | Low RoBs |
| Chen et.al/2004 | - | - | - | L | - | L | L | L | L | 5 | Moderate RoBs |
| Jiang et.al/2004 | - | L | L | L | - | L | L | L | L | 7 | Low RoBs |
| Du et.al/2003 | - | - | L | L | - | L | L | L | L | 6 | Moderate RoBs |
| Sun et.al/2003 | - | - | - | L | - | L | L | L | L | 5 | Moderate RoBs |
| Zhang et.al/2003 | - | L | - | - | - | L | L | L | L | 5 | Moderate RoBs |
| Wang et.al/2002 | - | L | L | - | - | L | L | L | L | 6 | Moderate RoBs |
| Meng et.al/1999 | - | - | L | - | - | - | L | L | L | 4 | High RoBs |
| Lin et.al/1999 | - | L | L | - | - | L | L | L | L | 6 | Moderate RoBs |
| Rong et.al/1999 | - | - | - | - | - | L | L | L | L | 4 | High RoBs |
| Tang et.al/1999 | - | L | L | - | - | - | L | L | L | 5 | Moderate RoBs |
| Hu et.al/1998 | - | - | - | - | - | - | L | L | L | 3 | High RoBs |
| Tang et.al/1998 | - | L | L | L | - | - | L | L | L | 6 | Moderate RoBs |
| WangL et.al/1997 | - | - | - | L | - | L | L | L | L | 5 | Moderate RoBs |
| WangH et.al/1997 | - | - | L | L | - | - | L | L | L | 5 | Moderate RoBs |
| Wan et.al/1993 | - | L | L | - | - | L | L | L | L | 6 | Moderate RoBs |
| Zhang et.al/1987 | - | - | - | - | - | L | L | L | L | 4 | High RoBs |
| Wang et.al/1985 | - | L | - | - | - | - | L | L | L | 4 | High RoBs |
| Zhou et.al/1984 | - | L | L | - | L | - | L | L | L | 6 | Moderate RoBs |
| Jiao et.al/1984 | - | L | L | L | - | - | L | L | L | 6 | Moderate RoBs |
| Bian et.al/1983 | - | - | L | - | - | - | L | L | L | 4 | High RoBs |
| Zhang et.al/2015 | - | - | - | - | - | L | L | L | L | 4 | High RoBs |
| Jin et.al/2014 | - | - | L | L | - | L | L | L | L | 6 | Moderate RoBs |
| Ko et.al/2009 | - | - | L | - | L | L | L | L | L | 6 | Moderate RoBs |
| Gau et.al/2005 | - | - | L | L | L | L | L | L | L | 7 | Low RoBs |
| Leung et.al/1996 | - | L | L | L | - | L | L | L | L | 7 | Low RoBs |
| Liu et.al/2014 | - | - | L | L | L | L | L | L | L | 7 | Low RoBs |

L: low risk.

If the criteria was met, a “L” would be recorded, or conversely, a “-”.
