# Supplementary material for: Prevalence of attention deficit/hyperactivity disorder among children and adolescents in China: a systematic review and meta-analysis
Source: BMC Psychiatry. 2017 Jan 19;17:32. doi: 10.1186/s12888-016-1187-9 (PMC5244567; doi:10.1186/s12888-016-1187-9)
Supplement: Additional file 4: — “Multiple comparisons of the prevalence estimates of ADHD reported by different sources of information”. (DOC 27 kb) [file 12888_2016_1187_MOESM4_ESM.doc]

**Additional file 4:**

Multiple comparisons of the prevalence estimates of ADHD reported by different sources of information

|  | Clinicians | |  | Subjects | |  | Parents | |  | Teachers | | |  | And rule | | |  | Unclear | |
| --- | --- | --- | --- | --- | --- | --- | --- | --- | --- | --- | --- | --- | --- | --- | --- | --- | --- | --- | --- |
|  | Q | *P* value |  | Q | *P* value |  | Q | *P* value |  | Q | *P* value | |  | Q | | *P* value |  | Q | *P* value |
| Subjects | 1.56 | 0.211 |  | - | - |  | 0.35 | 0.554 |  | 0.44 | | 0.507 |  | 0.42 | 0.518 | |  | 0.16 | 0.691 |
| Parents | 1.76 | 0.185 |  | 0.35 | 0.554 |  | - | - |  | 0.02 | | 0.877 |  | 0.09 | 0.758 | |  | 0.25 | 0.619 |
| Teachers | 0.85 | 0.376 |  | 0.44 | 0.507 |  | 0.02 | 0.877 |  | - | | - |  | 0.05 | 0.821 | |  | 0.37 | 0.543 |
| And rule | 0.02 | 0.887 |  | 0.42 | 0.518 |  | 0.09 | 0.758 |  | 0.05 | | 0.821 |  | - | - | |  | 0.25 | 0.618 |
| Unclear | 10.77 | 0.001 |  | 0.16 | 0.691 |  | 0.25 | 0.619 |  | 0.37 | | 0.543 |  | 0.25 | 0.618 | |  | - | - |
